# Safety of Aqueous Extract of *Calea ternifolia* Used in Mexican Traditional Medicine

**DOI:** 10.1155/2019/7478152

**Published:** 2019-12-26

**Authors:** Ma G. E. González-Yáñez, Catalina Rivas-Morales, María A. Oranday-Cárdenas, María J. Verde-Star, María A. Núñez-González, Eduardo Sanchez, Catalina Leos-Rivas

**Affiliations:** Universidad ‬Autónoma ‬de ‬Nuevo ‬León‬, Facultad ‬de ‬Ciencias ‬Biológicas. Av. ‬Universidad ‬s/n ‬Col. ‬Cd. Universitaria, CP 66455, San Nicolás de Los Garza, Nuevo León, Mexico

## Abstract

There is a trend to use medicinal plants for primary medical care or as dietary supplements; however, the safety of many of these plants has not been studied. The objective of this work was to determine the toxic effect of the aqueous extract of *Calea ternifolia* (*C. zacatechichi*), known popularly as “dream herb” *in vivo* and *in vitro* in order to validate its safety. *In vivo,* the extract had moderate toxicity on *A. salina. In vitro,* the extract induced eryptosis of 73% at a concentration of 100 *μ*g·mL^−1^ and it inhibited CYP3A by 99% at a concentration of 375 *μ*g/mL. After administering 8.5 mg/kg of *C. ternifolia* to rats, we found a reduction in platelets and leukocytes and an increase in urea and the liver enzymes alanine aminotransferase (ALT), aspartate aminotransferase (AST), and alkaline phosphatase (ALP). Histological analysis showed spongiform changes in the proximal tubules of renal tissue and a lymphoid infiltrate in liver tissue. This plant is used in the treatment of diabetes, and it is commercialized as a dietary supplement in several countries. Our results show renal and hepatic toxicity; therefore, more profound research on the toxicity of this plant is needed.

## 1. Introduction

Because of the current tendency to adopt a more “natural” lifestyle, there is currently great interest in herbal medicine, a medical system that searches for efficient and mildly or nontoxic therapeutic options. Medicinal plants have been used as a cultural inheritance in traditional medicine, but they require scientific validation [1], because they contain active substances that have biological activity and toxicity [2].

*Calea ternifolia* (syn. *C. zacatechichi*) (Astereaceae) is a plant native to Mexico and Central America, where it has a long tradition in indigenous culture [3]. It is also called the “dream herb” because it temporarily intensifies the clarity of dreams and significantly influences the central nervous system. For this, *C. ternifolia* is used in several countries, although it was placed on the prohibited plant list in Poland in 2013 [4]. A more recent study of the neuropharmacological action of the aqueous extract of *C. zacatechichi* indicates that it has insignificant neuropharmacological effects and also reduces the abdominal pain perception [5]. It is also traditionally used for the treatment of endocrine and gastrointestinal problems [6]. The plant is sold online as a dietary supplement for the treatment of diabetes due to its ability to cause hypoglycemia [7]. Although its mechanism of action is unknown, this activity can be due to compounds that have been isolated from the plant, such as flavonoids [8] and sesquiterpene lactones [9], which have some toxicity. Flavonoids cause deformation, osmotic fragility, and aggregation of erythrocytes [10]. Other flavonoids such as epigallocatechin produce toxicity in rat embryos [11], and quercetin, in addition to being able to capture free radicals and act as an antioxidant, can also carry out pro-oxidant reactions, exerting cytotoxic effects on cells and tissues [12]. The objective of this work was to determine the toxic effect *in vivo* and *in vitro* of the aqueous extract of *Calea ternifolia* (*C. zacatechichi)*, known popularly as “dream herb”. Although in this work several of the chemical components of *C. ternifolia* were identified, we only evaluated and demonstrated the toxicity of the complete aqueous extract.

## 2. Materials and Methods

### 2.1. Recollection and Identification of the Plant Material in Study

Plants were collected in 2012 at a farm plot in de Zacatecas, México (24°N 103°O). Botanical identification was carried out at the Universidad Autónoma Agraria Antonio Narro CRB-Agroecology Register 002/12. The aqueous extract of the leaves of the study plant was obtained and lyophilized (Labconco Corp., Kansas City, MO Serial No. 00627212F, Catalog No 7400030 Triad™).

The plant extract was analyzed by high-resolution liquid chromatography (HRLC). Phytochemical tests were carried out on the aqueous extract as described by Bruneton [13], and HRLC was performed on an Agilent model 1200 chromatograph (Agilent Technologies, Santa Clara, CA) with a quaternary pump and a diode array detector. Separation was performed with two Discovery® C18 columns 15 cm × 4.6 mm, 5 *μ*m (Supelco Analytical, Bellefonte, PA) connected in series with a 20 *μ*L loop. The mobile phase consisted of ammonium phosphate 50 mM adjusted to a pH of 2.6 with phosphoric acid (A), acetonitrile: ammonium phosphate 80 : 20 (B), and phosphoric acid 200 mM (C) to different gradients of A and B. Characterization of the extract compounds was carried out by comparing the retention times with standards, rutin (cat. no. 78095), chlorogenic acid (cat. no. 00500590), gallic acid (cat. no. G7384), syringic acid (cat. no. S6881), catechin (cat. no. 43412), epicatechin (cat. no. 68097), and quercetin (cat. no. 1592409) of Sigma-Aldrich, respectively, at a concentration of 1000 *μ*g·mL^−1^ 70 : 30 methanol:water [14].

### 2.2. Evaluation of the Biological Activity of the Extract in Human Erythrocytes

Human erythrocytes from healthy volunteer donors were obtained to perform the tests described below. All participants gave their written informed consent.

### 2.3. Erythrocyte Cytotoxicity of the Extract by Flow Cytometry

A sample of peripheral blood with the anticoagulant CDP/ADSOL™ was collected, and a complete blood count was determined on a Sysmex KX-21N Hematology Analyzer (Sysmex America, Inc., Lincolnshire, IL). A cell adjustment was made to 1 × 10^6^ mL^−1^ in 5 mL PBS (Cell Pack Phosphate Buffered Saline) and 1 *μ*L of whole blood in 5 mL HANKS′ balanced salt solution (Cat. H-2387 Sigma-Aldrich, Inc., St. Louis, MI). For flow cytometry, a FACSCalibur E4807 device (Becton Dickinson and Co., Franklin Lakes, NJ) was used. Erythrocytes were suspended at 0.4% [15] and/or 10^5^–10^6^ cells·mL^−1^. An eryptosis curve was run using rifampin 50 *μ*g/mL as a positive control and with the extract at concentrations of 1000, 500, 100, and 10 *μ*g/mL. The sample was centrifuged, and a pellet was obtained that was resuspended in 1 mL HANKS' solution; from this, 100 *μ*g/mL was taken and placed in a fluorescence-activated cell sorting (FACS) tube, 200 *μ*L of HANKS′ solution was added, and the sample was read by flow cytometry.

### 2.4. Flow Cytometry

For staining with Annexin V-FITC (Annexin V FITC, cat. 11-8005), the cells were washed with HANKS′ solution and subsequently resuspended in 1 mL of binding buffer at 1–5 × 10^6^·mL^−1^. From this suspension, 100 *μ*L was taken and 5 *μ*L of annexin V-fluorochrome conjugate was added and incubated at 22–28°C for 10–15 min. The cells were then washed in binding buffer and resuspended in 200 *μ*L of the buffer, kept in the dark at 2–8°C and analyzed by flow cytometry after 4 h.

### 2.5. Mean Corpuscular Volume (MCV)

Initially, the number of cells was adjusted to 1 × 10^6^ mL^−1^ in phosphate buffer (Cell Pack).

The extract at a concentration of 500 *μ*g was diluted with the cells 1 : 1 (extract : cells) and maintained at 20–25°C for 24 h; later this was read in the automated MCV device Sysmex KX-21 considering 80–97 fL as a normal value [16].

### 2.6. Thiobarbituric Acid/Malondialdehyde

The aqueous extract at a concentration of 500 *μ*g were assayed for thiobarbituric acid/malondialdehyde reactive substances (TBARS/MDA) to search for lipid peroxidation as a cell damage mechanism, following the method described by Dawn-Linsley et al. [17], using the TBARS Assay Kit item No. 10009055 from Cayman Chemical Co. (http://www.caymanchem.com).

### 2.7. Reactive Oxygen Species

For the quantification of reactive oxygen species, the Hydrogen Peroxide Cell-Based assay from Cayman Chemical Item Number 600050 was used. A total of 100 *μ*L of cells and 96 *μ*L of each extract (1500, 1000, 750, 500, 250, 100, 10, and 1 *μ*g/mL) were deposited in a 96-well plate and incubated in an atmosphere of 13–15% CO_2_ at 37°C for 24 h. They were then centrifuged, and the supernatant was transferred to a black plate adding 10 *μ*L of assay buffer to the extract and standard samples. Catalase was added to the positive control and the reactive enzyme solution to the standard, control, and extracts. Afterwards, they were incubated with constant orbital stirring for 20 min at a temperature of 20–25°C, and fluorescence was measured at 530–590 nm in an ELISA reader (WHY 101) [18].

### 2.8. Toxicity of Extracts against *Artemia salina*

The lethality assay on *A. salina* was performed as described by Leos-Rivas et al. [19], using the same toxicity criteria. Data were collected with IBM SPSS Statistics version 20 with probit regression to calculate LD_50_.

### 2.9. Histopathologic Damage to Male Wistar Rat Organs

#### 2.9.1. Experimentation Animals

This study was conducted with the approval of the Institutional Committee for the Care and Use of Laboratory Animals UAC, Mexico, file No. 25-03-14 and according to the provisions of the Official Mexican Standard NOM-062-ZOO-1999. Rats were housed in polycarbonate cages in conditions of humidity and with adequate temperature with 12-h light/dark cycles and with water and rat food (Prolab 2500) *ad libitum*.

#### 2.9.2. Animal Model of Histopathologic Damage

Twenty-five male Wistar rats (Rattus novergicus), 6–8 weeks old with an approximate weight of 200 g were used. The rats were divided into 5 groups of 5 rats each: three groups were given 1.25, 3.75, or 8.5 mg/kg of lyophilized plant extract resuspended in saline. Group 4 was given rifampin 8.5 mg/kg (positive control) and group 5, saline (negative control). Treatments were administered every 24 h for 7 d, and the doses were selected according to the oral reference dose for toxic noncarcinogenic substances [20, 21]. After the established time, blood samples were collected by cardiac puncture; one tube contained anticoagulant for hematological analysis and the other tube, without anticoagulant, was sent to biochemical analysis. The rats were immediately sacrificed by dislocation, and tissues and organs were collected for histopathological analysis in 10% buffered formalin.

#### 2.9.3. Histopathological Analysis of the Liver and Kidney

The organs preserved in 10% buffered formalin were placed on the dissecting plate. The main lobe of the liver and kidney were selected, and the tissue was placed in a formalin capsule for 7 h. The tissue was later placed in a Histokinet (Van Wissel-digital tissue processor) for 12 h with the capsule being ready and embedded in paraffin. Then, the paraffin cubes with the tissue were sectioned using a Spencer 820 Rotary Microtome. The fine slices of paraffin were placed in a flotation bath (Tissue Floating Bath XH-1001) at 52°C. They were deparaffinized for 15–30 min in a Fisher Scientific IsoTemp Incubator at 60°C. Subsequently, they were submerged in concentrated xylol for 10 min for the microscopic observation.

#### 2.9.4. Analysis of Parameters Evaluated in Male Wistar Rat Blood after Exposure to Treatments

The twenty-five experimental animal models were weighed before the assay and distributed in cages in 5 × 5 experimental units. After the established time, blood samples with an anticoagulant were collected and hematological parameters were analyzed using conventional methods.

#### 2.9.5. Induction of CYP450 by the Extract

CYP450 induction was performed by the kinetic enzyme method with the commercial kit VIVID® CYP450 RED (cat. no. P2856) [22]. Ketoconazole 10 *μ*M (as a positive inhibitor) (Sigma-Aldrich Cat. No. UC280), rifampin 10 *μ*M (Rifadin, as an inducer), and 2X buffer diluted with HPLC grade water were placed in a 96-well microplate with 100 *μ*L of different concentrations of the extract and 100 *μ*L of reaction buffer and as a target 200 *μ*L of buffer. A total of 50 *μ*L of 5 nM baculosomes was added to each well, the plate was incubated for 10 min at a temperature of 18–25°C, and a pre-reading was performed. The Vivid-NADP + substrate was prepared, and 10 *μ*L was added to each well and immediately read in an an ELISA (WHY 101) reader at 520 nm.

#### 2.9.6. Statistical Analysis

All the experiments were performed in triplicate, and the data were expressed as mean ± standard deviation (SD). The median lethal doses (LD_50_) were determined using a Probit regression with the SPSS version 20 software. The data were analyzed against negative control using comparison of pairs of treatment means (*t*-test), and statistical significance was established as *P*=0.01 for a confidence interval of 99%.

## 3. Results

**Figure 1:**
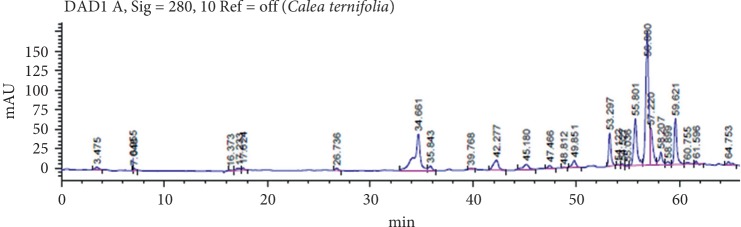
Chromatogram of the total extract of *C. ternifolia* by HPLC at a concentration of 1000 *μ*gmL^−1^, in a proportion 70 : 30 (methanol: water). The identification of compounds was carried out with the comparison of retention times of the standards.

The collected plants were identified at the UAAAN Torreon Unit with registration number CRB-Agroecology 002/012, obtaining a yield of 17% (p/p) of the aqueous extract. In the characterization of the *C. ternifolia* extract by HPLC (Figure 1), 30 compounds were found, and the retention time of three of them corresponded to the standards used: rutin (56.652), glycoside, quercetin (53.409), and epicatechin (34.661).

Table 1 shows the results obtained with the aqueous extract of *C. ternifolia* that caused an induction of eryptosis of 73% at a concentration of 100 *μ*g·mL^−1^; the positive control (rifampin) caused an induction of 76% at a concentration of 25 *μ*g·mL^−1^. There was a slight increase in the MCV with regard to normal values (>97 fL). The TBARS/MDA values were between 7.5 and 8.6 *μ*M greater than normal range (18.6–39.4 *μ*M). In the determination of H_2_O_2_, mean values were 14 *μ*M in comparison with 4.0 *μ*M for the control (without exposure to the extract). The aqueous extract of *C. ternifolia* had mild toxicity against *A. salina*.

**Figure 2:**
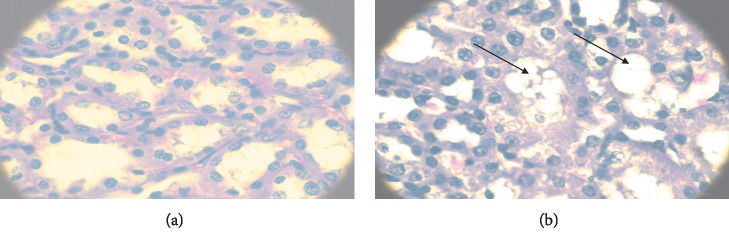
Comparison of histological sections of renal tissue of a healthy male Wistar rat (a) and a rat treated with *C. ternifolia* at 8.5 mg/kg (b) both with a haematoxylin and eosin stain. Spongiform changes are observed in the proximal tubules of the rat that received treatment (arrows).

**Figure 3:**
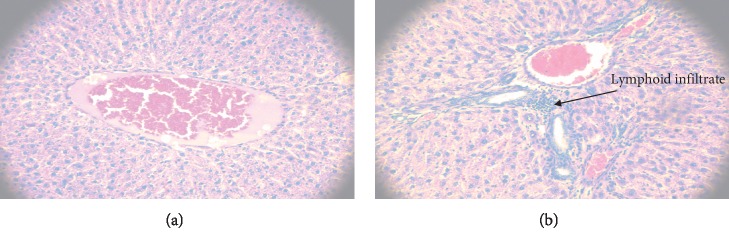
Comparison of histological sections of liver tissue of a healthy male Wistar rat (a) and a rat treated with *C. ternifolia* at 8.5 mg/kg (b) both with a haematoxylin and eosin stain. A lymphoid infiltrate is observed in the rat that received treatment.

In the experiment with the extract of *C. ternifolia* administered orally to male Wistar rats only at a dose of 8.5 mg/kg, spongiform changes were found in the proximal tubules of renal tissue (Figure 2) and a lymphoid infiltrate was present in liver tissue (Figure 3) after exposure to the extract in an assay performed in triplicate on the animal model.

The blood values of the animals under study before and after the administration of the aqueous extract of *C. ternifolia* at 8.5 mg/kg are shown in Table 2.

In these, platelets and leukocytes decreased and urea and liver enzymes (ALT, AST, and ALP) increased.  Table 3 shows the results of CYP3A4 inhibition of human baculosomes by the extract at different concentrations.

## 4. Discussion

The extract yield obtained from the plant was 1.7%. There are no previous reports although it has been found that the family Asteraceae has a yield of 8–10% when latex is extracted from its root [23] and aerial parts [24]. In the chemical study of C*. ternifolia*, rutin and quercetin were found, and there are previous reports of the presence of these flavonoids from Escandón-Rivera [9].

Some drugs such as ipratropium bromide and albuterol sulfate, which are used for obstructive lung disease, produce eryptosis [25]. Plant components that cause this same problem have been reported [26]. These natural components have antimicrobial, antiparasitic, anti-inflammatory, anticarcinogenic, cardiotonic, immunosuppressive, anti-infertility, neuroprotective, and antiatherosclerotic properties. One example of these is plumbagin (5-hydroxy-2-methyl-1.4-naphthoquinone). This phenolic compound induces eryptosis and/or apoptosis by mitochondrial depolarization, inhibiting the protein family Bcl-2. In this study, we found that C*. ternifolia* possesses phenolic compounds and that exposure of erythrocytes to the extract caused the induction of eryptosis. The polyphenolic components showed minimal hemolytic activity on human erythrocytes [27]. In this study, C*. ternifolia* extract showed a slight increase with regard to normal values.

In another study, the membrane of human erythrocytes exposed to radiation in the presence of quercetin was analyzed, and it was found that the concentration of TBARS increases in the presence of this compound, in addition to causing hemolysis [28]. In this study, something similar was observed with the presence of the C*. ternifolia* extract, which contains quercetin (increased values of TBARS/MDA).

A concentration greater or equal to 50 *μ*M of pure compounds is considered cytotoxic for animals, plants, and bacteria. However, in human blood, the cytotoxic concentration is ∼35 *μ*M [29]. Other authors report that it is near zero [30]. The aqueous extract of *C. ternifolia* used in this study has low toxicity and had weak toxicity against *A. salina* at 777 *μ*g·mL^−1^. According to the following criteria, DL_50_ > 1000 *μ*g·mL^−1^ is nontoxic; ≥500 and ≤1000 *μ*g·mL^−1^ is weakly toxic; and <500 *μ*g·mL^−1^ is toxic [31]. No previous studies of the toxicity of this plant were found.

There is research on the effects of phenolic compounds on cell function. Among these compounds, quercetin has been associated with oral toxicity [32]. The extract of *C. ternifolia* at a concentration of 8.5 mg/kg produced spongiform changes in the proximal tubules of renal tissue and the presence of a lymphoid infiltrate in liver tissue. Mossoba et al. [33], in a study of cell and mitochondrial function in human cells of the proximal tubule of the kidney (HK-2), reported elevated levels of nephrotoxicity biomarkers in *C. ternifolia*. Singh et al. [34], in studies of *E. alba* of the family Astereaceae, which contains flavonoids, reported similar results in the liver with adverse biochemical and histological effects at a concentration of 2,500 mg/kg. They observed that the oral administration of an aqueous extract of *E. alba* in female rats produced harmful effects on biochemical parameters and increased transaminase levels after liver damage due to induced membrane permeability; in a similar study alteration was found [33]. Later, the same author evaluated the aqueous extract of *Phyllanthus niruri* leaves and found liver damage at greater doses [35].

The group of enzymes that metabolize xenobiotics are called P450 cytochromes (CYPs), in particular, the enzyme CYP3A4. Inhibition of the activity of CYPs causes toxic effects in the body, and induction increases in its expression, affecting the metabolism, elimination and efficacy of drugs. Both processes can be evaluated and quantified with the modulation of CYP3A4 [36].

In recent studies, it has been found that the ligands of CYP450 interact with organic molecules [37]. In the study performed, the response of CYP450 to exposure of the study plant was observed.

The phytochemical components found in plants are organic substances that are metabolized in the liver with the possibility of inhibiting or inducing the expression of CYP3A4 in mammals. The characteristic of this enzyme is that the substrate occupying the active site leads to an inappropriate decrease in water and a decrease in the displacement of activated oxygen in water; thus, hydration is considered to be highly dependent on the ligand of some CYP isoforms. In addition, CYPs that metabolize drugs are characterized by a low degree of substrate selectivity [38]. CYP3A4 is involved in the oxidation of substrates and is responsible for the metabolism of drugs and phytochemical components, such as quercetin, a substance that is found in plants and is used in traditional medicine. This substance is considered an inhibitor of this enzyme, and also, it is necessary to evaluate the risks and benefits of its use as a natural product [32]. The function of these inhibitors leads to an increase in the effects that result from the consumption of their phytochemical components since they are not metabolized in the liver or in any other place in the body.

Phenolic components, such as rutin, epicatechin, and chlorogenic acid, have also been found in the study plant. As for quercetin, epicatechin, and rutin, it has been shown that they have the potential to attenuate radiation and cytogenic damage [39]. In this study, an assay was performed to determine the induction of CYP3A4, where activity was observed. Therefore, it can be considered that phenolic compounds act as antioxidants, but can also carry out pro-oxidant reactions, exerting cytotoxic effects on cells and tissues as well as the induction and inhibition of CYP450. It is also possible to consider that there is inhibition of CYP3A4 when there is exposure to the studied plant extract.

In this *in vitro* study, the aqueous extract of *C. ternifolia* showed moderate toxicity against *A. salina*, alterations in human erythrocytes, and inhibition of CYP3A4. *In vivo* results indicate high levels of biomarkers for nephrotoxicity and hepatotoxicity, possibly due to the chemical compounds, such as flavonoids, found in this study.

## 5. Conclusions

Based on the study conducted, *C. ternifolia* should be used for short periods of time because it has low toxicity against *A. salina* and produces undesirable effects in the liver and kidney of Winstar rats at 8.5 mg/kg. In traditional medicine, this plant is usually used for long periods of time, such as in the control of hiperglycemia. It is important to continue with the studies to identify the chemical components responsible for the biological activity, toxicity, and mechanism of action.

## Figures and Tables

**Table 1:** Biological and toxicological activities of the aqueous extract of *C. ternifolia*.

Plant/control	Eryptosis (%)	MCV (fL)	TBARS/MDA (*μ*Μ)	H_2_O_2_ (*μΜ*)	LD_50_*A. salina* (*μ*gmL^−1^)
*C. ternifolia*	73	100 ± 2.0	79 ± 1.5^*∗*^	14 ± 0.3^*∗*^	777 ± 8
Positive control	76	80 ± 1.9	65 ± 1.4	20 ± 0.4	<500
Negative control	12	100 ± 1.9	39 ± 1.4	4 ± 0.1	>1000

MCV: mean corpuscular volume; TBARS/MDA: thiobarbituric acid/malondialdehyde; H_2_O_2_: hydrogen peroxide; LD50: lethal dose 50%. SD: ±. ^*∗*^The extracts have significant difference against the negative control, *P*=0.01.

**Table 2:** Parameters evaluated in the blood of male Wistar rats after exposure to the different treatments after 7 d

Parameter	Rifampin 8.5 mg/kg (positive control)	H_2_O (negative control)	*C. ternifolia*		
			8.5 mg/kg	3.75 mg/kg	1.25 mg/kg
WBC (10^−3^)	5.0 ± 1.6	12.0 ± 1.9	4.3 ± 0.3	2.5 ± 0.9	3.9 ± 1.2
PLT (10^−3^)	294.3 ± 24.2	900.0 ± 3.9	418.0 ± 17.7	227 ± 10.2	193 ± 5.6
Urea (mg/dL)	69.5 ± 5.7	41.2 ± 5.9	57.8 ± 9.0	ND	ND
AST IU	85.0 ± 2.0	34.0 ± 2.3	546.0 ± 10.9	827 ± 4.7	212 ± 1.2
ALT IU	375.0 ± 1.5	50.0 ± 3.5	196.0 ± 21.1	548 ± 11.5	220 ± 9.6
F ALK IU	376.0 ± 5.0	33.0 ± 3.8	386.0 ± 5.7	697 ± 6.3	457 ± 4.9

WBC: leukocytes, PLT: platelets, ALT: alanine aminotransferase, AST: aspartate aminotransferase, ALP: alkaline phosphatase, IU: international units, ND: no determinate, and SD: ±. All the treatments have significant difference against the negative control, *P*=0.01.

**Table 3:** Inhibition of CYP3A4 by the aqueous extract of *C. ternifolia* and controls on human baculosomes.

Plant or control	% inhibition of CYP3A4		
	1500 *μ*g/mL	750 *μ*g/mL	375 *μ*g/mL
*C. ternifolia*	59 ± 3	88 ± 4^*∗*^	99 ± 5^*∗*^
Rifampin 10 *μ*M (inductor)		56 ± 2	
Ketoconazol 10 *μ*M (positive inhibitor)		95 ± 5	

SD: ±. ^*∗*^The extracts have significant difference against the negative control, *P*=0.01.

## Data Availability

The data used to support the findings of this study are included within the article.
